# Quantitative genetic analysis reveals potential to breed for improved white clover growth in symbiosis with nitrogen-fixing *Rhizobium* bacteria

**DOI:** 10.3389/fpls.2022.953400

**Published:** 2022-09-20

**Authors:** Sean K. Weith, M. Z. Zulfi Jahufer, Rainer W. Hofmann, Craig B. Anderson, Dongwen Luo, O. Grace Ehoche, Greig Cousins, E. Eirian Jones, Ross A. Ballard, Andrew G. Griffiths

**Affiliations:** ^1^AgResearch, Grasslands Research Centre, Palmerston North, New Zealand; ^2^Faculty of Agriculture and Life Sciences, Lincoln University, Lincoln, New Zealand; ^3^PGG Wrightson Seeds Ltd., Grasslands Research Centre, Palmerston North, New Zealand; ^4^South Australian Research and Development Institute (SARDI), Adelaide, SA, Australia

**Keywords:** *Trifolium repens* (white clover), quantitative genetics and breeding, *Rhizobium leguminosarum* bv. *trifolii* TA1, plant-microbe interactions, genetic gain, nitrogen fixation, symbiosis

## Abstract

White clover (*Trifolium repens*) is integral to mixed pastures in New Zealand and temperate agriculture globally. It provides quality feed and a sustainable source of plant-available nitrogen (N) *via* N-fixation through symbiosis with soil-dwelling *Rhizobium* bacteria. Improvement of N-fixation in white clover is a route to enhancing sustainability of temperate pasture production. Focussing on seedling growth critical for crop establishment and performance, a population of 120 half-sibling white clover families was assessed with either N-supplementation or N-fixation *via* inoculation with a commercial *Rhizobium* strain (TA1). Quantitative genetic analysis identified significant (*p* < 0.05) family additive genetic variance for Shoot and Root Dry Matter (DM) and Symbiotic Potential (SP), and Root to Shoot ratio. Estimated narrow-sense heritabilities for above-ground symbiotic traits were moderate (0.24–0.33), and the strong (*r* ≥ 0.97) genetic correlation between Shoot and Root DM indicated strong pleiotropy or close linkage. The moderate (*r* = 0.47) phenotypic correlation between Shoot DM under symbiosis vs. under N-supplementation suggested plant growth with mineral-N was not a strong predictor of symbiotic performance. At 5% among-family selection pressure, predicted genetic gains per selection cycle of 19 and 17% for symbiotic traits Shoot DM and Shoot SP, respectively, highlighted opportunities for improved early seedling establishment and growth under symbiosis. Single and multi-trait selection methods, including a Smith-Hazel index focussing on an ideotype of high Shoot DM and Shoot SP, showed commonality of top-ranked families among traits. This study provides a platform for proof-of-concept crosses to breed for enhanced seedling growth under *Rhizobium* symbiosis and is informative for other legume crops.

## Introduction

Over the last century, plant breeding has played a key role in the genetic improvement of the agronomic performance of white clover (*Trifolium repens* L.). In addition to its contribution to the sward as a high-quality and palatable forage, white clover forms root nodules where atmospheric dinitrogen (N_2_) is fixed to provide bio-available nitrogen (N) through symbiosis with soil-borne *Rhizobium* bacteria (Fred et al., 2002; Graham and Vance, 2003). As an example of changes in N application, New Zealand’s pastoral industry has historically relied heavily upon biologically fixed N inputs. In recent years, however, the application of N fertilisers on dairy farms has increased six-fold to an average of 120 kg N per hectare between 1991 and 2009 (Glassey et al., 2013). This intensification has led to unsustainable N fertiliser over-application, increasing risks of nitrate leaching into groundwater and waterways (Andrews and Lea, 2013). Furthermore, the manufacture of synthetic N fertilisers *via* the Haber-Bӧsch process is estimated to consume ~2% of the world’s primary energy supply annually (Erisman et al., 2008), releasing large amounts of carbon dioxide thus increasing the global carbon footprint. Consequently, there is now significant pressure on pastoral industries world-wide to increase sustainability through the reduction of N fertiliser use.

Improvement of N-fixation in white clover is a route to enhancing pasture production sustainability and has implications for temperate agriculture globally. N-fixation *via* legume-*Rhizobium* symbiosis is, however, underpinned by a complex inter-kingdom dialogue where both the host plant and the *Rhizobium* strain contribute genetic variation which influences nodulation and N-fixation (Mytton, 1983). Sequenced genomes of *Rhizobium leguminosarum* bv *trifolii* strains associated with *Trifolium* species (Reeve et al., 2010; Terpolilli et al., 2014; Delestre et al., 2015; Cavassim et al., 2020; Perry et al., 2021) provide a platform for further elucidation of this symbiosis, and variation in *Rhizobium* nodulation genes has been shown to influence host specificity and competitive nodule competency (Ferguson et al., 2020). While the white clover genome itself has been sequenced (Griffiths et al., 2019), there is little insight into the role genetic variation in the white clover host has in driving effective symbiotic outcomes. To date, several studies have shown that the variation in N-fixation capacities of individuals within populations of white clover and other legume species is quantitative and polygenically controlled (Gibson, 1962; Connolly et al., 1969; El-Sherbeeny et al., 1977).

Over the past several decades, inoculation of white clover seed *via* a coating with superior strains of *Rhizobium* has been a commonly utilised practice in New Zealand (Lowther and Kerr, 2011). This ensured that the seedling taproot, which sustains the plant for the first 12–18 months, is inoculated with high-performing *Rhizobium* to achieve prompt and effective legume N-fixation during plant establishment (Lowther and Kerr, 2011). Establishment of vigorous seedlings is key to the production of a successful crop or pasture, as exemplified by Ehoche et al. (2022) who showed that effective white clover establishment and growth in the first year of a mixed sward cattle-grazed field trial had a strong positive correlation with clover performance in subsequent years. This provides a breeding target focussed on improving interaction with *Rhizobium*, hence N-fixation, at the seedling stage with either commercial inoculant or soil-borne wild-type strains. For plant breeders to improve N-fixation in white clover, the genetic variation of symbiotic traits needs to be estimated and utilised in cultivar development programmes to maximise genetic gain. Determining and quantifying the additive genetic variance, the component of the phenotypic variation inherited from parents to offspring (Falconer and Mackay, 1996), is instructive to plant breeders for improving genetic gain in trait performance (Acquaah, 2012). The total phenotypic expression of any individual in a population is a combination of its genotypic value (G), environmental effect (E) and a complex interaction of both (Falconer and Mackay, 1996). Breeding for N-fixation is complicated further by the plant genotype by *Rhizobium* genotype interaction. To date, white clover breeding has predominantly targeted agronomic traits such as dry matter yield and persistence, with a strong emphasis on broad adaptation by including genotype by environmental interactions (Woodfield and Caradus, 1990; Chapman et al., 1995; Widdup and Barrett, 2011; Jahufer et al., 2013; Ehoche et al., 2022). The complexity of studying the *Rhizobium* symbiosis at an agronomic level may explain why there are few studies reporting estimates of additive genetic variation for traits associated with N-fixation in legumes, and these have often focussed on nodulation (number and size of nodules) (Arrenddell et al., 1985; Greder et al., 1986; Herridge and Rose, 1994; Crush and Caradus, 1995) rather than biomass accumulation. This highlights the challenges faced when breeding cultivars with enhanced *Rhizobium* symbiosis as a target trait.

To investigate and determine the quantitative genetic parameters of the white clover-*Rhizobium* interaction at the seedling stage, a genetically structured breeding population comprising 120 half-sibling (half-sib) families was assessed for symbiotic traits. This population structure is commonly used for breeding in outcrossing species, such as white clover, and facilitates integration of the quantitative genetic estimates derived from this study into a breeding programme. The objectives of this study were to: a) estimate the magnitude of additive genetic variation among these half-sib families for key traits associated with N-fixation using the commercial *Rhizobium* inoculant TA1; b) identify high N-fixing families as parents for crossing to improve symbiotic traits; and c) determine the predicted rate of genetic gain based on deterministic modelling using among half-sib family selection methodology. This will give insight into the potential of the clover-*Rhizobium* symbiosis as a target trait for improved seedling establishment for reduced-input agriculture.

## Materials and methods

### Plant material, *Rhizobium* strain and experimental design

The half-sib family populations were developed in 2012 from 31 clonal cuttings from top performing breeding lines that were polycrossed to form an F_1_ synthetic population of 141 half-sib families, as described (Ehoche et al., 2022). Briefly, a balanced bulk representation of these 141 F_1_ half-sib families was created by selecting an equal number of seeds from each family. From this bulk, a random sample of 141 seeds were grown and polycrossed to generate 141 F_2_ half-sib families, three of which were removed due to producing insufficient seed. Two individuals were then selected randomly from each of the remaining 137 half-sib families, making a total of 274 individuals that were poly-crossed to generate 274 F_3_ families. Crossing was performed using pre-washed wild bumble bees (*Bombus* spp.) in a single bee-proof tunnel house, and the plants were repositioned randomly at regular time intervals to mitigate against pollen bias. The population of 120 half-sib families used in this study was a random sample taken from the 274 F_3_ generation half-sib families.

White clover half-sib families were assessed in two controlled environment growth rooms using a two environmental replicate randomised split plot experimental design generated with DeltaGen software^1^ (Jahufer and Luo, 2018). In this design the containers and the quadrants within the containers (described below) were the plots and sub-plots, respectively. Each environmental replicate was housed in a separate growth room and consisted of 60 4.4 L containers containing 500 g of Grade 3 vermiculite which were saturated with 2.0 L of low-N McKnight’s solution (McKnight, 1949). Treatments consisted of half-sib families either: 1) inoculated with a pure culture of the commercial *Rhizobium leguminosarum* bv. *trifolii* strain TA1 or 2) supplied with mineral N in the form of ammonium nitrate (NH_4_NO_3_) (positive control), as described below. Each container was divided into quadrants, and each quadrant was planted with one of four half-sib families that were assigned randomly to treatment container and quadrant within each replicate using a row and column experimental design. Seeds from the half-sib families were lightly scarified using sandpaper and surface-sterilised following a method described by Drew et al. (2012), placed on filter paper moistened with autoclaved deionised water, stratified in the dark for 48 h at 4°C for uniform germination, then incubated in the dark for 12–24 h at 25°C until the radicles were ~4 mm long. A total of 16 seedlings per half-sib family were transplanted individually, using flame-sterilised tweezers, into the appropriate quadrant at 1 × 1 cm spacing in four rows of four seedlings. Across the two environmental replicates, therefore, each half-sib family was represented by 32 seedlings (biological replicates). Four additional containers similarly divided into quadrants and neither inoculated with *Rhizobium* nor provided with mineral N (negative control), were included per environmental replicate. This was to allow seed N reserves to be considered in the calculation of symbiotic potential post-harvest and indicated the absence of *Rhizobium* contamination among experimental treatments. This negative control container was planted with a balanced bulked mixed sample of all half-sib families. Each quadrant had 16 individuals, hence 64 balanced bulk seedlings per negative control container, 256 per replicate and 512 in the experiment to represent the mean genetics of the half-sib families. The trial plan is detailed in Supplementary Figure S1.

The *Rhizobium leguminosarum* bv. *trifolii* strain TA1 was used to screen the symbiotic performances of half-sib families based on its known effective N-fixation capacity and use as a commercial inoculant strain in New Zealand and Australia. Cultures of the TA1 strain were sourced directly from glycerol stocks stored at −80°C and plated initially onto yeast mannitol agar (YMA) slopes. The TA1 strain was sub-cultured onto new YMA slopes and grown in an incubator for four days at 25°C in continuous darkness. For white clover seedling inoculation, the TA1 cultures were suspended in 500 ml of sterile deionised water to provide a concentration of 1 × 10^7^ cells mL^−1^. Each white clover seedling was inoculated one day after sowing by applying a 0.5 ml aliquot of *Rhizobium* suspension. The number of cells applied was confirmed by spreading a 1 × 10^−7^ dilution of the inoculant suspension on YMA plates and counting colony forming units after four days incubation at 25°C in continuous darkness.

Positive control half-sib family individuals received approximately 3.1 ml aliquots of 15 mmol L^−1^ NH_4_NO_3_ (1.2 g L^−1^) with the first dose applied nine days after sowing and weekly thereafter (200 ml container^−1^) for three weeks. The initial aliquot was timed such that the positive control seedlings received N when N-fixation most likely began in *Rhizobium*-inoculated seedlings, based on preliminary vermiculite assays when white clover individuals inoculated with TA1 had one or more pink/effective nodules (Mytton, 1973).

### Plant growth and maintenance

The two controlled environment growth rooms had a photoperiod of 16 h light and 8 h dark at a constant day and night temperature of ~22°C. Photosynthetically active radiation (PAR), was provided by four Apollo 600 W light emitting diode (LED) (seegree®) light banks with two variants (6 and 12 bulbs) and two 4 × 100 W Cob integrated LED (Easy Grow Ltd) light banks per growth room. Radiation corresponding to far-red (660 to 730 nm), and infrared (700 nm to 1,000 nm) was provided by six 100 W/240 V R95 Spotone incandescent lamps (Philips) per growth room. Each treatment container was watered to approximately 80% of its initial moistened weight at sowing (~2000 g) at intervals of 1 to 3 days to maintain plant water status.

### Phenotypic measurements

Shoot dry matter (DM) and Root DM were determined after 35 days of post-inoculation growth. Shoots and roots of each plant were separated at the hypocotyl, dried for 72 h in paper bags at 60°C and weighed using a four-decimal place precision balance. The Root to Shoot ratio (RSR) of each white clover individual was calculated post-harvest by dividing the amount of Root DM by the amount of Shoot DM. Shoot and Root DM values were used to calculate symbiotic potential (SP). The SP allowed the impact of biological N-fixation of white clover half-sib families to be compared. The SP describes the proportion of biomass generated through symbiosis relative to supplementation with mineral N (positive control) achieved by an individual of a white clover half-sib family. This calculation also adjusts for the influence on DM production caused by seed weight and seed N reserves by accounting for DM production from uninoculated plants. Symbiotic potential of each half-sib plant was calculated using Equation 1 as described by Drew et al. (2012) with modifications:


(1) $$SP\left(\%\right)=\frac{\begin{array}{l} \mathrm{Shoot}\;DM\;\left(\mathrm{inoculated plant}\right)-\\ \mathrm{Shoot}\;DM\;\left(\mathrm{uninoculated plants}\right) \end{array}}{\begin{array}{l} \mathrm{Shoot}\;DM\;\left(\mathrm{positive}\;N\;\mathrm{control plants}\right)-\\ \mathrm{Shoot}\;DM\left(\mathrm{uninoculated plants}\right) \end{array}}X\;100$$

Where: SP, symbiotic potential; DM, dry matter (mg plant^−1^); inoculated plant, an individual from a specific half-sib family inoculated with *Rhizobium*; uninoculated plants, the mean Shoot DM value (mg plant^−1^) of all individuals from a random subsample of a balanced bulk of all half-sib families grown without *Rhizobium* or supplemental N; positive N control plants, the mean Shoot DM value (mg plant^−1^) of individuals from the specific half-sib family grown without *Rhizobium* but with supplemental N.

To calculate Root SP, the Shoot DM values in Equation 1 were replaced with Root DM.

### Quantitative genetic analysis

A linear model analysis was performed using the residual maximum likelihood (REML) procedure (Patterson and Thompson, 1971; Harville, 1977) to estimate the family additive genetic variance ($\sigma_{A}^{2}$) associated with symbiotic related traits among the 120 half-sib families using DeltaGen (Jahufer and Luo, 2018) based on Equation 2:


(2) $$Y_{ijkl}=\mu +f_{i}+R_{j}+{\left(fR\right)}_{ij}+r_{kj}+c_{lj}+\varepsilon_{ijkl}$$

where: $Y_{\mathrm{ijkl}}$, is the value of a trait measured from half-sib family i in row k and column l of environmental replicate j, and i = 1,….$,n_{f}$, j = 1,….$,n_{R}$, k = 1,…$n_{r}$, l = 1,….$,n_{c}$, where f,R,r,c and ε are half-sib families, environmental replicate, row, column and residual effects, respectively; μ is the overall mean; $f_{i}$ is the random effect of the half-sib family *i* and modelled as coming from a normal distribution with the given mean and variance, $N\left(0,I\sigma_{f}^{2}\right)$; $R_{j}$is the random effect of environmental replicate j, $\sim N\left(0,I\sigma_{R}^{2}\right)$; ${\left(fR\right)}_{ij}$is the random effect of the interaction between half-sib family *i* and environmental replicate j, ~$N\left(0,I\sigma_{fR}^{2}\right)$; $r_{kj}$ is the random effect of row *k* in environmental replicate *j*, $\sim N\left(0,I\sigma_{r}^{2}\right)$; $c_{lj}$ is the random effect of column *l* in environmental replicate *j*,$\sim N\left(0,I\sigma_{c}^{2}\right)\text{;}$ and $\varepsilon_{\mathrm{ijkl}}$ is the residual effect of family *i* in environmental replicate *j* in row *k* and column *l*,$\sim N\left(0,I\sigma_{\varepsilon }^{2}\right)$.

For each trait, half-sib family means were adjusted for spatial variation effects within the experimental design by generating best linear unbiased predictor (BLUP) values. Initial data analyses using the split-plot design in the statistical model found that the variance components associated with the sub-plots (eg half-sib family × sub-plot interactions) were not significant with variances of zero. Therefore, a model was used from which the sub-plot structure was excluded. The BLUPs were generated from REML analysis of all traits as described by White and Hodge (1989) for each half-sib family treatment combination using DeltaGen. REML analysis was conducted with the half-sib families as random effects. Initial analyses with the environmental replicates as fixed effects in a linear mixed model produced the same outcomes as when the replicates were random effects. Consequently, the analysis was performed using linear models with no fixed effects.

Pairwise Pearson correlation coefficients analysis was performed to determine the linear relationship of the traits Shoot DM, Root DM and RSR as well as Shoot symbiotic potential (SP) and Root SP. The Pearson correlation coefficient for each trait was generated from the BLUP values of white clover half-sib families which had been separated into treatments using the ‘cor’ and ‘corr.test’ functions in the R package ‘psych’ (Revelle, 2017). The corresponding value of p for testing each correlation coefficient difference from zero (significance when *p* ≤ 0.05) were generated using the R package ‘car’ (Fox and Weisberg, 2018). The correlation coefficients and *P* values for each symbiotic trait-treatment combination were used to construct a pairwise correlation matrix which was visualised by generating a correlation plot using the R package ‘corrplot’ (Wei and Simko, 2021).

Narrow-sense heritability ($h_{n}^{2}$) values among half-sib families for each trait were estimated on a half-sib family mean basis for each treatment separately using a model proposed by Nyquist and Baker (1991) in DeltaGen (Jahufer and Luo, 2018). Estimation of $h_{n}^{2}$ for the symbiotic traits was derived using the variance components from linear mixed model (Equation 2) in Equation 3:


(3) $$h_{n}^{2}=\frac{\sigma_{A}^{2}}{\sigma_{A}^{2}+\frac{\sigma_{AR}^{2}}{n_{R}}+\frac{\sigma_{\varepsilon }^{2}}{n_{R}}}$$

where: $h_{n}^{2}$ is the narrow-sense heritability; $\sigma_{A}^{2}$, family additive genetic variance component; $\sigma_{AR}^{2}$, family additive-by-environmental replicate interaction variance component; $\sigma_{\varepsilon }^{2}$, residual variance; and $n_{R}$, number of environmental replicates. As the analysis uses a structured half-sib population, the estimated variance components are additive (Falconer and Mackay, 1996) rather than both additive and dominance/non-additive effects, hence heritability estimates are narrow-sense.

### Trait correlations

A multi-trait analysis of variance (MANOVA) was performed to determine the genetic correlation between the traits within treatments. This correlation is the family additive genetic covariance between traits normalized by the geometric mean of individual trait variances and provides an estimate of genetic factors shared between traits *via* pleiotropy or linkage. MANOVA was conducted in DeltaGen using a random linear model shown in Equation 2 to generate variance–covariance and genetic correlation coefficients for the association between the traits.

Pattern analysis was conducted using a combination of cluster analysis and principal component analysis (PCA) to assess the grouping of half-sib families within each treatment based on expression of all traits. The aim was to identify and group half-sib families with relatively similar trait performance (DeLacy et al., 1996). Pattern analysis was conducted using the half-sib family-by-symbiotic trait BLUP matrix generated from REML analysis as described. Cluster analysis was performed using a hierarchical agglomerative classification procedure (Wishart, 1969) with squared Euclidean distance as a measure of dissimilarity (Burr, 1968, 1970) and the Hartigan clustering algorithm (Hartigan, 1975) to identify the optimum number of groups. The PCA was performed as described by Jolliffe (2002) using DeltaGen. Groups identified by cluster analysis were assigned individual colours and then superimposed onto a PCA biplot. The correlation structure among symbiotic traits was indicated by the angles between directional vectors.

### Estimated genetic gain, selection indices and correlated response to selection

Genetic gain (ΔG) was predicted only for those symbiotic traits with significant (*p* ≤ 0.05) family additive genetic variance among the 120 half-sib families. To model predicted response to selection, based on among half-sib family selection, the ΔG per selection cycle was estimated using 5, 10 and 20% selection pressures (*p*), equating to selection intensities (k) of 2.06, 1.76, and 1.4, respectively. The breeders’ equation used, as described by Casler and Brummer (2008) for among half-sib family selection in obligate outcrossing forage crops, is outlined in Equation 4:


(4) $$\Delta G_{HSF}=k_{f}c\;\frac{\frac{1}{4}\;\sigma_{A}^{2}}{\sigma_{PF}}$$

Where: $\Delta G_{HSF}$, genetic gain using among half-sib family selection, hereafter referred to as ΔG; $k_{f}$, is the standardised selection differential among half-sib families; c, the parental control factor (c = 0.5 for half-sib families);$\sigma_{A}^{2}$, family additive genetic variance and $\sigma_{PF}$, the phenotypic standard deviation among families.

The Smith-Hazel (SH) index described by Smith (1936) and Hazel (1943), was used in this study to develop index coefficients to facilitate selection on a multi-trait basis using DeltaGen based on Equation 5:

(5) $$\left[b\right]={\left[P\right]}^{-1}\left[A\right]\left[w\right]$$

Where: $\left[b\right]\text{,}$ the vector of index coefficients; ${\left[P\right]}^{-1}$ and $\left[A\right]\text{,}$ phenotypic and additive genetic variances and covariances, respectively; and $\left[w\right]\text{,}$ the vector of economic weightings.

This generates an index based on quantitative genetic parameters to enable co-selection of multiple traits with a single value which permits the identification of half-sib families with, in the case of this study, high Shoot DM and Shoot SP. The economic weightings were substituted by desired genetic gain (Jahufer and Luo, 2018). Equal weightings (1.0) were given to both traits.

The correlated selection of traits enables selection for a secondary (target) trait X while also obtaining a correlated response from a primary trait Y. In this study, this was investigated to assess the possibility of identifying and selecting for plants with high biomass under symbiosis that also have large SP. Correlated response to selection ($\Delta G_{c}$) for the symbiotic traits Shoot DM and Shoot SP was estimated using an equation proposed by Falconer and Mackay (1996) using Equation 6:


(6) $$CR_{Y-HS}=k_{f}ch_{X}h_{Y}r_{Axy}\sigma_{PY}$$

Where: $CR_{Y-HS}$, correlated response to selection ($\Delta G_{c}$) in primary trait Y which selecting for a secondary (or target) trait $X;k_{f}$, among half-sib family selection pressure; c, among half-sib family parental control (c = 0.5 for half-sib families); $h_{X}$ and $h_{Y}$, square root of the narrow-sense heritabilities for symbiotic traits X and Y, respectively; $r_{Axy}$, genetic correlation between traits X and Y; and $\sigma_{PY}$, among family phenotypic standard deviation of symbiotic trait Y.

## Results

### Symbiotic traits had significant variance components and moderate heritability

Best linear unbiased predictors (BLUPs) of the symbiotic traits showed variation among the 120 half-sib families. When *Rhizobium* strain TA1 was the provider of nitrogen *via* symbiosis, mean Shoot dry matter (DM) was 11.4 mg plant^−1^ with a five-fold difference between the highest and lowest half-sib family (Table 1). Root DM exhibited a similar difference, although the Root to Shoot ratio (RSR) had an approximately two-fold range among the families. Calculated Shoot symbiotic potential (SP) had a mean of 40% ranging from 12 to 85%, indicating that with *Rhizobium* some half-sib families produced as much as 85% of the DM generated with mineral N (Table 1). By contrast, the Root SP ranged up to 140% indicating Root DM was sometimes greater in the *Rhizobium* treatment than when supplemented with N. A feature of the trial was the variation among the 120 HS families in Shoot DM generated *via* symbiosis and Shoot SP (Supplementary Figure S2). These families were derived from elite material. The RSR showed one response to reduced N input was to partition more biomass to root growth, as demonstrated by mean RSR increasing from 0.38 to 0.48 and 0.82 for the negative control (no *Rhizobium*; no mineral N), *Rhizobium* treatment and when supplemented with mineral N, respectively (Table 1). The negative control data measured the biomass generated when N was sourced primarily from the seed components. Additionally, cross-contamination of *Rhizobia* among treatments, which would confound analysis, was not a feature of these trials as there was no evidence of nodulation in either the negative or N-supplemented controls. These results provide strong evidence that the primary source of N in the TA1 *Rhizobium* treatment was N-fixation *via* symbiosis.

**Table 1:** Trait Best Linear Unbiased Predictor (BLUP) values, variance components and narrow-sense heritabilities for 120 white clover half-sib families either inoculated with *Rhizobium leguminosarum* bv. *trifolii* strain TA1 or grown with mineral nitrogen and no *Rhizobium*, or a balanced bulk of 120 half-sib families grown with no *Rhizobium* nor mineral nitrogen.

Trait	Shoot DM (mg)	Shoot SP (%)	Root DM (mg)	Root SP (%)	RSR (mg/mg)
Inoculated with *Rhizobium* strain TA1					
Range	4.4–23	12–85%	2.1–9.6	10–140%	0.38–0.61
x̅	11.4	40%	5.03	48%	0.48
$\sigma_{Α}^{2}$	13.5 ± 2.5*	0.01 ± 0.004*	2.2 ± 0.44*	0.04 ± 0.009*	0.001 ± 0.0009*
$\sigma_{ΑR}^{2}$	4.7 ± 1.3*	0.01 ± 0.002*	0.9 ± 0.2*	0.01 ± 0.005*	0.003 ± 0.001*
$\sigma_{\varepsilon }^{2}$	49.6 ± 1.1	0.09 ± 0.002	10.2 ± 0.2	0.2 ± 0.005	0.04 ± 0.001
$h_{n}^{2}$	0.33 ± 0.04	0.24 ± 0.04	0.28 ± 0.04	0.26 ± 0.03	0.05 ± 0.02
No *Rhizobium*, supplemented with mineral nitrogen					
Range	7.8–42.1	n/a	4.1–15.5	n/a	0.29–0.54
x̅	25.1	n/a	9.00	n/a	0.38
$\sigma_{Α}^{2}$	23.3 ± 6.5*	n/a	3.4 ± 0.8*	n/a	0.0002 ± 0.0005
$\sigma_{ΑR}^{2}$	23.6 ± 6.5*	n/a	2.6 ± 0.7*	n/a	0.003 ± 0.0007*
$\sigma_{\varepsilon }^{2}$	220.6 ± 5.2	n/a	30.7 ± 0.7	n/a	0.02 ± 0.0005
$h_{n}^{2}$	0.16 ± 0.03	n/a	0.17 ± 0.03	n/a	0.01 ± 0.04
No *Rhizobium*, no supplementation with mineral nitrogen					
Range	2.13–2.18	n/a	1.53–1.65	n/a	0.82–0.84
x̅	2.15	n/a	1.59	n/a	0.82

*x̅*, mean; $\sigma_{Α}^{2}\text{,}$ estimated half-sib family additive genetic variance; $\sigma_{ΑR}^{2}\text{,}$ family × replicate interaction variance; $\sigma_{\varepsilon }^{2}\text{,}$ residual error variance; ±, ± standard error; $h_{n}^{2}\text{,}$ narrow-sense heritability; DM, dry matter plant^−1^; SP, symbiotic potential plant^−1^ indicating the clover biomass produced with *Rhizobium* as a proportion of the biomass produced with mineral nitrogen; RSR, root to shoot ratio; n/a, not applicable as SP is not a feature of the mineral nitrogen-supplemented traits.

*significant family additive genetic variation (*P* ≤ 0.05) (This estimate equals ¼ of total additive genetic variation as the analysis has been performed with a half-sib family structure).

Variance components showed significant (*p* ≤ 0.05) family additive genetic variation ($\sigma_{A}^{2}$) among the 120 half-sib families inoculated with the *Rhizobium* strain TA1 for all the symbiotic traits (Table 1). While the family × environmental replicate interaction components ($\sigma_{AR}^{2}$) were significant (*p* ≤ 0.05) for these traits, these were equal to the family additive genetic variation component ($\sigma_{A}^{2}$) for Shoot SP and lower than Shoot DM, Root DM and SP (Table 1). This suggests while significant, there was little re-ranking of families among the two growth rooms for these traits. Conversely, although small, the family × environmental replicate interaction variance component for RSR was greater than family variance, indicating more family re-ranking among environmental replicates for this trait compared to the others. The estimates for narrow-sense heritabilities for the symbiotic traits measured on a half-sib family basis ranged from low to moderate with 0.05 for RSR and up to 0.33 for Shoot DM.

For the N-supplemented treatments, the mean Shoot DM BLUP value for the 120 half-sib families was approximately twice that of the *Rhizobium*-only treatments (25.7 and 11.4 mg plant^−1^, respectively), as was Root DM (Table 1). Similar to the *Rhizobium* treatment, the N treatment had an approximately five-fold difference between the highest and lowest half-sib Shoot DM BLUPs, whereas the Root DM had approximately four-fold range of values (Table 1). While the RSR mean was lower compared with the *Rhizobium* treatment (0.3 and 0.48 mg/mg, respectively), the range differential was similar (Table 1). There was significant (*p* ≤ 0.05) family additive genetic variation among the 120 half-sib families grown with mineral nitrogen for Shoot and Root DM but not RSR (Table 1). Like the *Rhizobium* treatment, the family × environmental replicate interaction variance components were significant (*p* ≤ 0.05) for the measured traits (Table 1). Similarly, the interaction variance components were equal or less than the family variance for Shoot and Root DM, indicating little family re-ranking for these traits among growth rooms. Residual error variance components were greater than family and family × environmental replicate interactions components, and larger than the *Rhizobium* treatment for Shoot and Root DM (Table 1). Narrow-sense heritability estimates for the traits measured on a half-sib family basis were low and ranged from 0.01 for RSR to 0.17 for Root DM.

### Genetic correlations among traits

Multi-trait analysis of variance (MANOVA) genetic correlation coefficients for five traits expressed by the 120 half-sib families when inoculated with the *Rhizobium* strain TA1 are presented in Table 2. These coefficients estimate the level of genetic control in common for two different traits (Falconer and Mackay, 1996), and ranged from weak to strong positive or negative between shoot and root traits for DM as well as symbiotic potential (SP). Notable genetic correlations included strong positive coefficients between Shoot and Root DM, and between Shoot and Root SP. Shoot and Root DM each had moderate positive genetic correlation coefficients (~0.60) with Shoot and Root SP. Conversely, RSR had weak negative genetic correlation coefficients with Shoot and Root DM (−0.33 and −0.12, respectively). When the 120 half-sib families were grown with supplemental mineral nitrogen and no *Rhizobium*, there was a very strong positive genetic correlation between Shoot and Root DM, and a weak to moderate negative association between RSR and Root or Shoot DM, respectively (Table 2). While the correlations among most traits with N supplementation were similar to those with rhizobia, there was, however, no correlation between RSR and Root DM (Table 2).

**Table 2:** Multi-trait analysis of variance (MANOVA) genetic correlation coefficient values between five traits of 120 white clover half-sib families inoculated with the *Rhizobium leguminosarum* bv. *trifolii* strain TA1, and between three traits when supplemented with mineral nitrogen and no *Rhizobium.*

	Rh-Shoot DM (mg)	Rh-Shoot SP (%)	Rh-Root DM (mg)	Rh-Root SP (%)	N-Shoot DM (mg)	N-Root DM (mg)
Rh-Shoot SP (%)	0.62					
Rh-Root DM (mg)	0.97	0.63				
Rh-Root SP (%)	0.53	0.85	0.60			
Rh-RSR (mg/mg)	−0.33	0.01	−0.12	0.32		
N-Root DM (mg)	n/a	n/a	n/a	n/a	0.94	
N-RSR (mg/mg)	n/a	n/a	n/a	n/a	−0.39	−0.05

Rh, *Rhizobium* inoculation treatment; N, supplemented with mineral nitrogen treatment with no *Rhizobium*; DM, dry matter plant^−1^; SP, symbiotic potential plant^−1^ indicating the biomass produced with *Rhizobium* as a proportion of the biomass produced when supplemented with mineral nitrogen; RSR, root to shoot ratio; n/a, not applicable as multi-trait correlations were made within treatments.

### Pattern analysis described correlations among treatments

**Figure 1:**
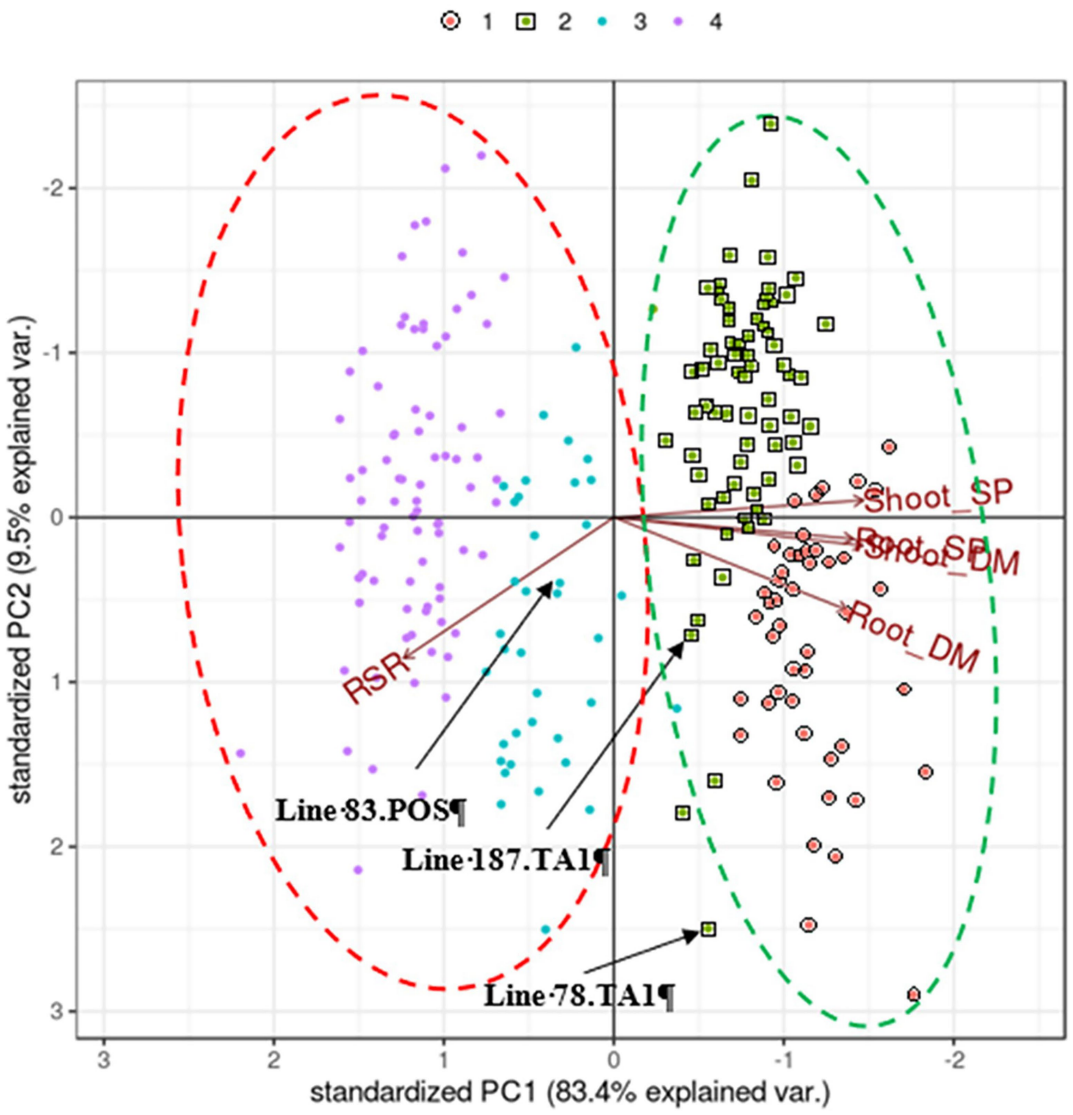
Biplot generated by pattern analysis using best linear unbiased predictor (BLUP) adjusted means of 120 white clover half-sib families assessed with two treatments: *Rhizobium leguminosarum* bv. *trifolii* strain TA1; and No *Rhizobium*, nitrogen-supplemented (positive control; POS). Plant responses associated with PCA dimensions 1 and 2 are Shoot DM (dry matter plant^−1^); Root DM; Shoot SP (symbiotic potential plant^−1^); Root SP; RSR (root to shoot ratio plant^−1^). Colours (top of figure) indicate half-sib family membership to pattern analysis Cluster groups (1–4). The coloured dashed circles indicate manually constructed trend lines for treatment clusters; red, indicates TA1 Cluster groups; green, indicates No *Rhizobium*, nitrogen-supplemented treatment Cluster groups. Half-sib families of interest (eg Line 83.POS) are indicated for discussion in the text.

Principal component analysis of the BLUP matrix of traits measured in 120 half-sib families grown with either mineral N (positive controls) or inoculated with the *Rhizobium* strain TA1 showed that components PC1 and PC2 explained 83.4 and 10% of the variation, respectively (Figure 1).

There was a positive association (directional vectors <90° relative to each other) between Shoot DM and Root DM as well as the symbiotic traits Shoot SP and Root SP (Figure 1) which reflected that of the genetic correlation analysis (Tables 2 and 3). Similarly, the RSR was negatively associated (directional vectors >90° relative to each other) with Shoot DM, Shoot SP, Root DM and Root SP. The associations were supported further by phenotypic correlations coefficients among these traits where there were moderate to very strong positive correlations (0.59–0.92) among Shoot and Root DM and SP (Supplementary Table S1). Like the pattern analysis, RSR was negatively correlated with the other traits. Correlation strength was reduced when comparing traits between the two treatments (Supplementary Table S1).

**Table 3:** Number of white clover half-sib families assigned to each of four cluster groups generated from the pattern analysis of five traits of 120 half-sib families inoculated with the *Rhizobium leguminosarum* bv. *trifolii* strain TA1 or grown with supplemental mineral nitrogen (N-supplemented).

	Treatment										
	TA1						N-supplemented			
Cluster group	No. of lines (n = 120)	Shoot DM (mg)	Shoot SP (%)	Root DM (mg)	Root SP (%)	RSR (mg/mg)	No. of lines (n = 120)	Shoot DM (mg)	Root DM (mg)	RSR (mg/mg)
1 (n = 47)	0	np	np	np	np	np	47	30.2	11.1	0.39
2 (n = 74)	2	20.4	84%	8.8	121%	0.48	72	22.7	7.8	0.37
3 (n = 37)	36	15.4	55%	6.7	68%	0.48	1	9.2	4.1	0.52
4 (n = 82)	82	9.4	33%	4.2	37%	0.49	0	np	np	np

For each cluster group, the mean BLUP values (best linear unbiased predictor adjusted means) for the measured traits for the members of each treatment in each cluster are presented. DM, dry matter plant^−1^; SP, symbiotic potential plant^−1^ indicating the clover biomass produced with *Rhizobium* as a proportion of the biomass produced with mineral nitrogen; RSR, root to shoot ratio plant^−1^; np, not present, where half-sib families from a particular treatment are not present in that cluster.

SP is not a feature of the mineral nitrogen-supplemented traits.

There was a trend separating clusters based on treatment as the half-sib families grown under mineral N showed above average expression of Shoot and Root DM (Figure 1). By comparison, most *Rhizobium*-inoculated half-sib families had reduced expression for Shoot and Root DM and the corresponding SPs but had increased RSR. Within these trends, four Cluster groups were identified (Figure 1; Table 3) which were separate except for some overlap between Clusters 2 and 3. Cluster 1 consisted of half-sib families grown under mineral N treatment. The half-sib families within this Cluster group displayed above average expression for Shoot DM and Root DM with mean BLUPs of 30.2 and 11 mg plant^−1^, respectively (Table 3). Cluster 2 consisted mostly of half-sib families grown under mineral N (mean Shoot DM = 22.7 mg plant^−1^), but also contained two half-sib families (lines 78 and 187) that had been inoculated with *Rhizobium* strain TA1 (mean Shoot DM = 20.3 mg plant^−1^). These two TA1-inoculated half-sib families had performed similarly to mineral N treated families and displayed above average expression for Shoot and Root DM and SP, and RSR. The mean Root SP was 121%, indicating these two half-sib families generated more symbiotic root biomass relative to the N supplemented root biomass. Clusters 3 and 4 consisted mostly of half-sib families inoculated with the *Rhizobium* strain TA1, highlighting the performances of these families when N is derived from symbiosis. Cluster 3 had one half-sib family (line 83) from the mineral N treatment (Shoot DM = 9.2 mg plant^−1^) indicating this family performed similarly to, or worse than, half-sib families inoculated with *Rhizobium* (mean Shoot DM = 15.4 mg plant^−1^). Features of Clusters 3 and 4 were below-average expression, relative to the mineral N treatment, of all traits except RSR. This indicated increased partitioning into root biomass above shoot biomass when half-sib families were reliant on the symbiosis for N supply.

### Predicted genetic gain per cycle shows potential for symbiotic trait improvement

**Figure 2:**
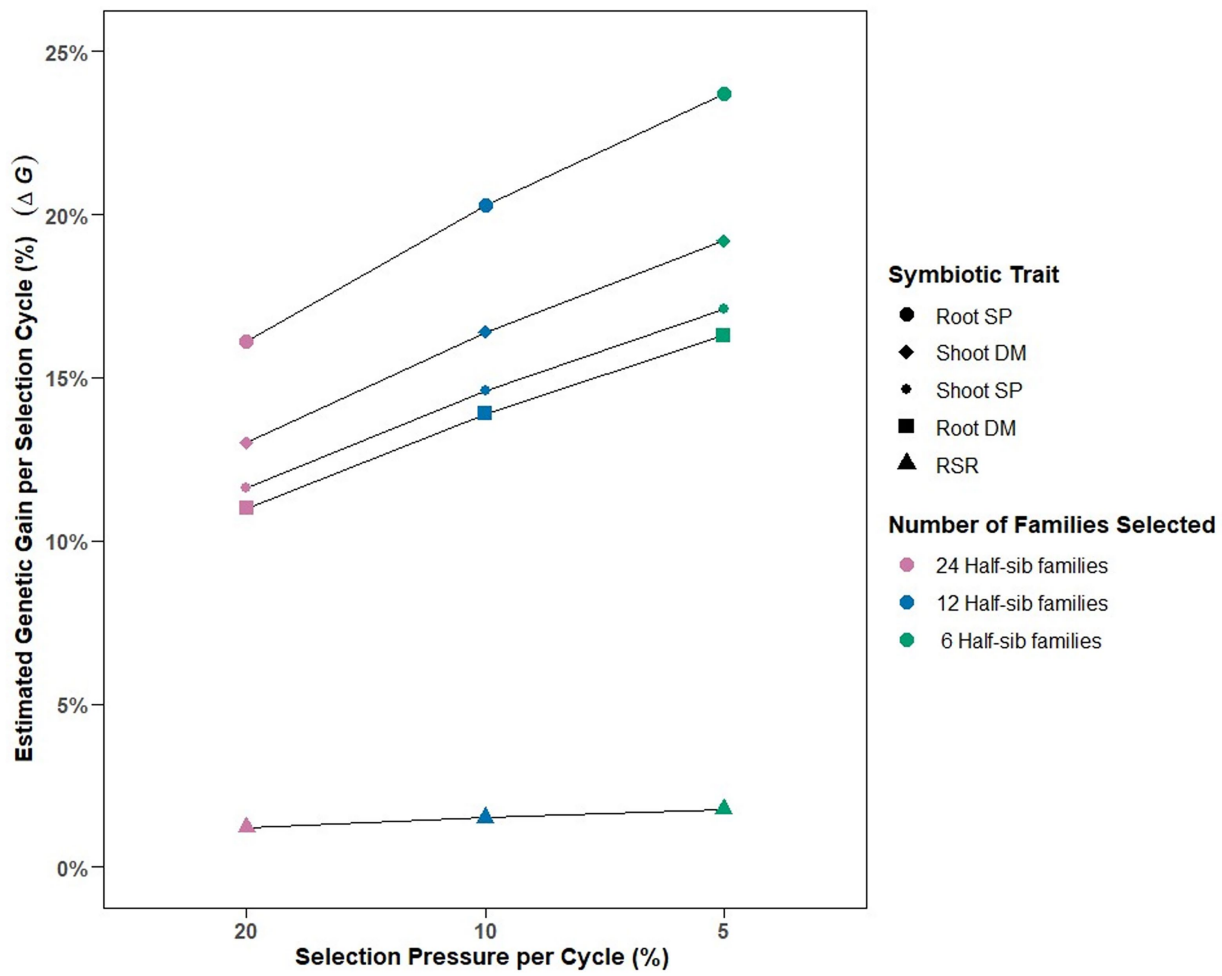
Predicted estimated genetic gains (ΔG) relative to absolute means and number of half-sib families selected for among-family selection pressures of 20% (n = 24 families), 10% (n = 12 families) and 5% (n = 6 families) per selection cycle for the five symbiotic traits assessed in 120 white clover half-sib families inoculated with the *Rhizobium leguminosarum* bv. *trifolii* strain TA1. SP, symbiotic potential plant^−1^; DM, dry matter plant^−1^; RSR, root to shoot ratio plant^−1^.

The calculated variance components enabled deterministic prediction of genetic gain (%ΔG) per selection cycle for each symbiotic trait under different selection pressures (Figure 2). Selection pressures of 20, 10 and 5%, equating to selection intensities (k) of 2.06, 1.76, and 1.4, respectively, were derived by identifying 24, 12 and 6, respectively, of the top performing 120 half-sib families for each symbiotic trait. Predicted genetic gain per cycle increased as selection pressure increased from 20 to 5%, with Root SP having the greatest %ΔG response (16–23%), followed by Shoot DM (13–19%), Shoot SP (11–17%), and Root DM (11–16%) (Figure 2). RSR had the lowest predicted genetic gain across the selection pressures (1.2–1.7%).

### Selections based on single trait or multi-trait selection index

Important symbiotic breeding objectives for clover production include Shoot DM and Shoot SP, a calculated trait which indicates the proportion of biomass produced *via* symbiosis relative to that produced with mineral N. These traits can be selected for individually or concurrently in a multi-trait selection index. A desirable target would be to have high SP packaged with a high Shoot DM plant, therefore aligning high biomass with efficient use of symbiotically-derived N. Selecting for these traits independently has drawbacks as SP alone does not reflect total plant biomass since small plants may have a high SP. To this effect, a Smith-Hazel multi-trait selection index for Shoot DM and SP was calculated by integrating phenotypic correlation and family additive genetic variation components for Shoot DM and Shoot SP. The mean values of the top ranked half-sib lines identified at 5% among-family selection pressure were 20.6 mg plant^−1^, 75% and 12.0 for Shoot DM BLUPs, Shoot SP and a Smith-Hazel index (SH), respectively (Supplementary Table S2). As the pressure reduced from 10 to 20%, the number of families selected increased. This reduced the mean trait value of the half-sib families selected for Shoot DM BLUPs, Shoot SP and SH, which were 19.04 mg, 69.2%, 11.09, and 17.4 mg, 62.1%, 10.3, respectively (Supplementary Table S2).

**Figure 3:**
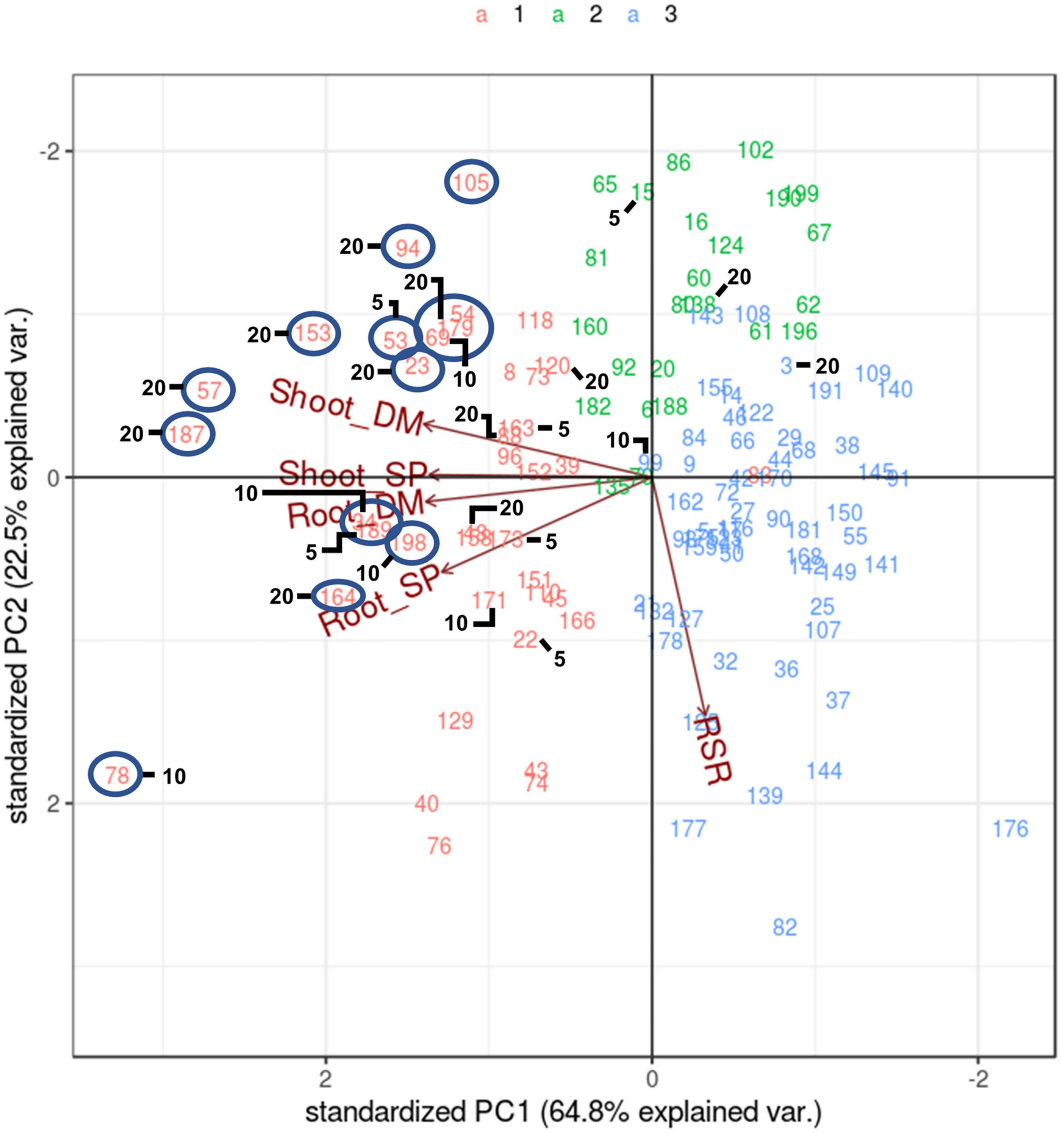
Biplot generated by pattern analysis using best linear unbiased predictor (BLUP) adjusted means of 120 white clover half-sib families inoculated with *Rhizobium leguminosarum* bv. *trifolii* strain TA1. Plant responses associated with principal component (PCA) dimensions 1 and 2 are Shoot DM (dry matter plant^−1^); Root DM; Shoot SP (symbiotic potential plant^−1^); Root SP; RSR (root to shoot ratio plant^−1^). Numbers and colours (top of figure) indicate half-sib family line number and membership to pattern analysis Cluster groups (1–3), respectively. The circles indicate half-sibling families showing above average expression for traits Shoot DM and SP that may be candidate families for selection. Numbers 5, 10 and 20 denote half-sib families identified using a Smith-Hazel multi-trait selection index based on Shoot DM and SP at 5% (n = 6 families), 10% (n = 12 families) and 20% (n = 24 families) selection pressures, respectively. Combining 5 and 10 yields the 10% selection pressure, whereas 5, 10 and 20 combined are the families at 20% selection pressure.

Another method of multi-trait selection is pattern analysis whereby half-sib families are distributed based on performance across all measured traits. They are then selected according to biplot placement relative to vectors of the traits of interest, rather than using a specific index value. A pattern analysis of the symbiosis treatment alone (Figure 3) reflected that of the analysis with both *Rhizobium* and supplemented N biplot (Figure 1), except that there was little to no correlation between RSR and the other traits (directional vectors are ~90° relative to each other). Half-sib families showing higher expression for both Shoot DM and Shoot SP that may be selected for crossing were identified and were members of Cluster 1 (Figure 3). Most half-sib families identified using the SH index based on Shoot DM and SP across the selection pressures were located in Cluster 1 and in the biplot associated with higher Shoot and Root DM and SP. They also aligned with many candidates identified using pattern analysis (Figure 3). One exception was half-sib family 54 which was positioned near 179 and 69 in the biplot (Figure 3). It was not selected with the SH index but did contribute to individual trait selections for Shoot DM and Shoot SP at 20% selection pressure (Supplementary Table S2). While there was commonality in half-sib families selected between the two multi-trait selection methods, pattern analysis is a graphical diagnostic tool based on association among traits. Family selection is, therefore, influenced by the breeder’s interpretation of the biplot. The SH index, however, allows ranking of families for multi-trait performance using a numerical index. This empirical approach enables more effective comparison with the single trait selections and was, therefore, the multi-trait selection of choice in this study.

**Figure 4:**
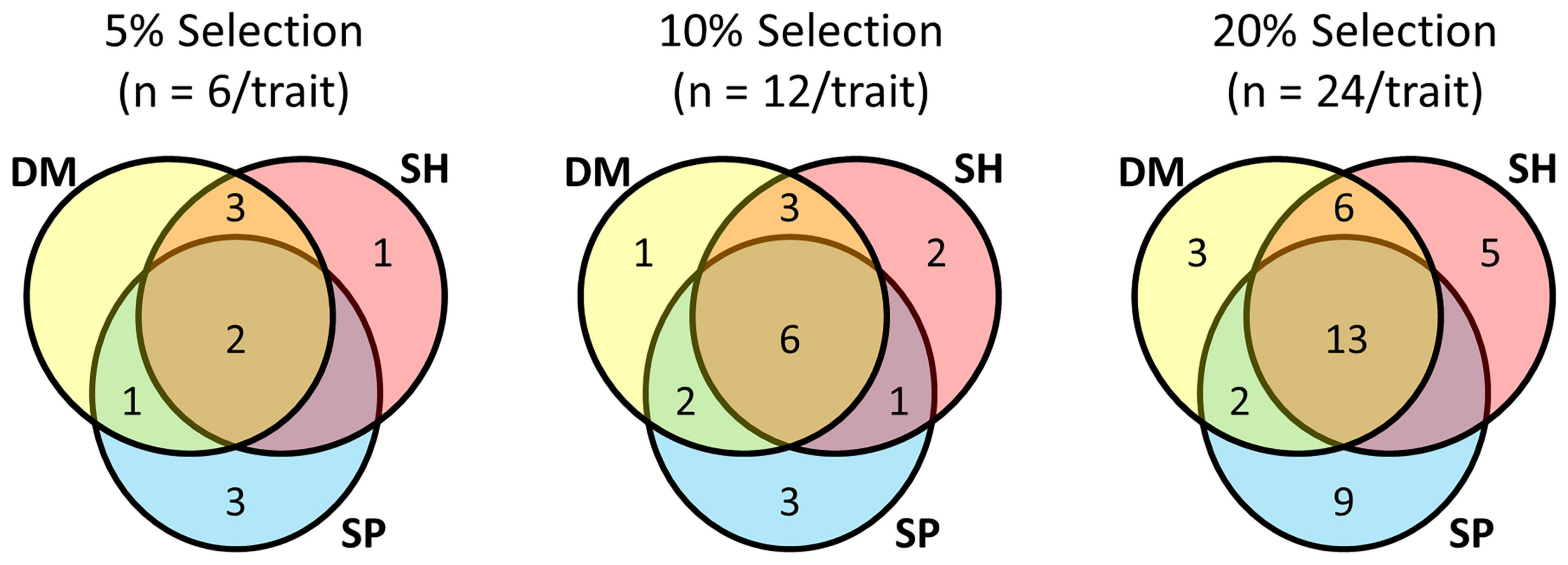
Commonality of half-sib families among selections made for symbiotic traits Shoot Dry Matter (DM), Shoot symbiotic potential (SP), and a Smith-Hazel (SH) multi-trait selection index for both Shoot DM and SP at three selection pressures. From a total of 120 half-sib families inoculated with the *Rhizobium leguminosarum* bv. *trifolii* strain TA1, 5% selection pressure selected the top ranked six families for each trait or SH index, based on trait BLUPs or SH index, 10% comprised the top ranked 12 families, and 20% the top ranked 24 families for each trait or SH index. BLUP and Smith-Hazel index values are presented in Supplementary Table S2.

Focussing on the multi-trait SH index and comparing it with half-sib families ranked by their BLUPs for individual traits (Shoot DM, Shoot SP), there was commonality among the top performing lines at the different selection pressures (Figure 4; Supplementary Table S2). When selection pressure decreased from 5 to 20%, the half-sib families shared among each trait selection increased, as did the number of half-sib families unique to each trait selection. Irrespective of selection pressure, there were more families common to Shoot DM and SH index selections than to Shoot SP and SH index selections. At the highest selection pressure (5%), five of the six families for each trait were in both Shoot DM and SH index selections, and only two common to Shoot SP and the SH index selections. Shoot SP had the greatest number of half-sib families not represented in the other selections which ranged from three to nine as the selection pressure decreased (Figure 4). The variation for Shoot DM and Shoot SP within each half-sib family as well as the families selected at each selection pressure is shown in Supplementary Figure S2. These half-sib families would provide the basis of proof-of-concept crosses for among-family selection to breed for improved white clover-*Rhizobium* symbiotic traits.

### Correlated response to selection

To gain further insight into the relationship between key white clover agronomic symbiotic traits and how these may be deployed in a breeding programme, the correlated response to selection for Shoot DM and Shoot SP was investigated. The correlated response to selection ($\Delta G_{c}$) assesses how selection for a secondary, or target, trait influences a correlated response in a primary trait. As the focus in this study was to develop plants with high biomass under symbiosis that also have high symbiotic potential, Shoot DM and Shoot SP were the traits of interest. Trait data including narrow-sense heritability and genetic correlation, described above, were used to estimate correlated response at different selection pressures.

Correlated responses to selection at 20, 10 and 5% selection pressures resulted in a range of predicted $\Delta G_{c}$ for the Shoot SP and Shoot DM under symbiosis depending upon which trait was selected as the secondary, or target, trait (Table 4). In general, the correlated response to selection was similar for both traits and increased with selection pressure. With Shoot DM as the secondary (target) trait, the correlated response from Shoot SP resulted in predicted $\Delta G_{c}$ of 9 to 13% relative to the Shoot SP absolute mean as selection pressure increased from 20 to 5% (Table 4). This was very similar to when Shoot SP was the target trait, except the correlated response from Shoot DM was lower (7–11%). These responses suggest that co-selection for these two traits may facilitate development of a plant ideotype with both enhanced Shoot DM and Shoot SP under symbiosis.

**Table 4:** Predicted correlated response ($\Delta G_{c}$) to selection per cycle for symbiotic traits Shoot DM and SP.

Secondary (target) trait	Primary trait	Primary trait absolute mean	Pressure per selection cycle	$\Delta G_{c}$ of primary trait relative to its absolute mean
Shoot DM (mg)	Shoot SP (%)	40%	20%	3.5% (8.7%)
			10%	4.4% (11.0%)
			5%	5.2% (12.9%)
Shoot SP (%)	Shoot DM (mg)	11.4 mg	20%	0.8 mg (7.3%)
			10%	1.1 mg (9.7%)
			5%	1.2 mg (10.7%)

Shoot DM (dry matter plant^−1^; mg) and Shoot SP (symbiotic potential plant^−1^; %) in white clover. When selecting for a secondary (target) trait at different selection pressures (20, 15 and 5%), the correlated response of the primary trait per selection cycle, based on data from the 120 white clover half-sib families inoculated with the *Rhizobium leguminosarum* bv. *trifolii* strain TA1, is described.

## Discussion

### Variance components and narrow-sense heritability

Previous white clover studies have focussed on family additive genetic variation of simple and complex agronomic traits (Jahufer et al., 1994, 2009, 2013; Ehoche et al., 2022) rather than symbiotic traits themselves. The significant family additive genetic variation and the moderate narrow-sense heritabilities ($h_{n}^{2}$) for shoot and root biomass traits provide a novel dataset to explore symbiosis for enhanced N-fixation and has breeding implications for this important forage legume. While the family × environmental replicate (growth room) interaction variance components were significant for all traits, they were either equal to or less than the family additive genetic variances for Shoot and Root dry matter (DM) and symbiotic potential (SP), particularly for the *Rhizobium*-inoculated treatment. This suggests stability of family performance across the environments and, therefore, performance in one growth room was a good predictor of performance in the other.

Heritability estimates for white clover symbiotic traits have thus far been associated predominately with nodulation features such as nodule size and mass (Jones and Burrows, 1969; Mytton and Rys, 1985), which does not necessarily translate into biomass. The current study reports $h_{n}^{2}$ which describes the family additive genetic variation as a proportion of the phenotypic variation, and provides an estimate of genetic variation that may be manipulated by breeders (Falconer and Mackay, 1996). The $h_{n}^{2}$ was low (0.24) to moderate (0.33) for Shoot SP and Shoot DM, respectively, under symbiosis. Traits with moderate $h_{n}^{2}$ may be improved faster than traits with lower heritabilities (Nyquist and Baker, 1991), indicating that symbiosis-derived Shoot DM has greater potential for improvement than Shoot SP. In white clover, $h_{n}^{2}$ for agronomic traits including internode length, stolon number, leaf size, and dry matter yield have ranged from 0.09 to 0.99 (Woodfield and Caradus, 1990; Annicchiarico et al., 1999; Ehoche et al., 2022). The higher values were for traits that are less complex and less prone to phenotyping variation, such as leaf size (0.99; Annicchiarico et al. (1999)). The low to moderate symbiotic $h_{n}^{2}$ reported in the current study likely reflects the complexity of the legume × *Rhizobium* symbiotic relationship. A previous white clover symbiosis study focusing on nodule traits reported a broad-sense heritability of 0.48 for the number and mass of nodules for a population of the white clover cultivar ‘S100’ (Mytton and Rys, 1985). Broad-sense heritability, however, is inflated relative to $h_{n}^{2}$ as it comprises both additive and non-additive genetic variation components (Falconer and Mackay, 1996). Heritabilities of traits such as nodule number, nodule mass and nodule nitrogenase activity tend to be greater than for plant biomass under symbiosis. This may reflect that the conversion from nodule activity to plant growth has complex intermediate steps and that nodulation itself is a simpler trait or indicates that it is earlier in the symbiosis process.

In a recent study performed with combinations of *Rhizobium* strains and genotypes from white clover cultivars, Moeskjaer et al. (2021) reported low to moderate broad-sense heritabilities, on an individual plant phenotype level, ranging from 0.23 to 0.32 for a set of symbiotic traits including: growth per day; growth during a specific window post-inoculation; and corrections of these for initial plant size. When using a genomic relationship matrix to derive $h_{n}^{2}$ based on clover genotype means, the values for these traits ranged from 0.24 to 0.27, when corrected for initial plant size (Moeskjaer et al., 2021). This aligns with the symbiotic yield $h_{n}^{2}$ values described in the current study. The trial reported by Moeskjaer et al. (2021) assessed stolon cuttings, which represent the mature clover nodal-rooting growth stage, from a range of genetic material. By contrast, the current study focussed on seedlings from a half-sib family structure growing on their initial taproot. The similarity in $h_{n}^{2}$ values for symbiotic traits across diverse white clover populations and plant stages suggests consistency in the plant genetic components driving the *Rhizobium* interaction, further supporting the feasibility of these traits as breeding targets.

In summary, these results highlight the potential within the white clover half-sib population for the genetic improvement of symbiotic traits. Combined with a moderate $h_{n}^{2}$, there is a possibility for improvement of valuable traits such as Shoot DM under symbiosis through either mass or individual phenotypic selection approaches. However, greater gains would be expected *via* family-based selection strategies which can lift genetic gain markedly for lower heritability traits (Casler and Brummer, 2008; Jahufer et al., 2021).

### Genetic correlations highlight commonality of genetic mechanisms among symbiotic traits

Genetic correlation among traits provides an estimate of commonality of genetic mechanisms which may be due to pleiotropy or linkage (Falconer and Mackay, 1996). These correlations also give insight into likelihood of improvement of one trait when selecting for another. To date, genetic correlations have been reported between white clover agronomic traits such as stolon density, branching and thickness (Jahufer et al., 1995; Ehoche et al., 2022), and seeds per pod, potential floret size and potential seed yield (Jahufer and Gawler, 2000). The genetic correlation coefficients for the symbiotic traits in the current study ranged from weak to strong, and positive or negative in pairwise associations, and reflected the phenotypic correlations and trait biplot generated from pattern analysis. The strongest genetic correlations in TA1-inoculated populations were between Shoot and Root DM (0.97) and between Shoot and Root SP (0.85), indicating that in this population the genetic mechanisms for these correlated shoot and root traits are either highly pleiotropic or linked. Importantly, while root trait data are often described in symbiosis trials, they are time and labour-intensive, hence resource-intensive to collect (Mytton, 1983; Hardarson and Danso, 1993; Azam and Farooq, 2003). The strong genetic and phenotypic correlations between Root and Shoot DM under symbiosis suggest that selection for increased Shoot DM will have a concomitant response in Root DM. If the breeding goal in this population is focused on above-ground biomass under symbiosis, then root biomass will likely be co-selected, and its measurement adds little additional value.

By contrast, the moderate genetic correlation (0.62) between Shoot DM and Shoot SP indicates fewer genetic mechanisms in common than Shoot and Root DM as there were instances when families had high Shoot DM and low Shoot SP, and vice versa. This is understandable as Shoot SP is somewhat independent of total biomass. For example, a plant or family with low biomass under symbiosis may have a high SP if the biomass of the N-supplemented control is also low. Conversely, a plant or family with a large symbiotic biomass may have a low SP if the N-control is markedly greater. Even so, the moderate genetic correlation between Shoot DM and Shoot SP suggests potential for co-selecting these two traits to develop highly productive plants with high SP. There were, however, moderate negative genetic and phenotypic correlations between Shoot DM and root to shoot ratio (RSR). This suggests antagonistic genetic mechanisms and increased partitioning into root biomass relative to shoot biomass when half-sib families were grown in a N-reduced environment. This phenomenon was particularly evident in the no N, no *Rhizobium* controls. From a field breeding perspective, the moderate positive phenotypic correlation (0.47) between Shoot DMs with either supplemental mineral N or under symbiosis indicates that growth under mineral N was not an accurate predictor of symbiotic performance. This highlights that N-supplementation may confound analysis of symbiosis-based traits.

Overall, the genetic correlation data from this half-sib family study, in tandem with the phenotypic correlations, show potential to breed for an ideotype combining both high biomass and high SP under symbiosis using a commercial *Rhizobium* strain.

### Symbiotic traits had high levels of predicted genetic gain per cycle

Genetic gain calculations across the selection pressures showed notable per cycle estimates with up to 19, 17 and 23% for symbiotic Shoot DM, Shoot SP and Root SP, respectively. There is, therefore, opportunity for marked improvement in DM production of seedlings reliant on symbiotic N-fixation in this population. The predicted genetic gain also reflected the $h_{n}^{2}$ values for these traits, which were moderate (0.24–0.33). At present, there are few studies reporting estimated genetic gains for symbiotic traits in white clover or other legume species. An unpublished study cited by Woodfield (1999) reported that, using ^15^N analysis across three trial sites, the genetic gain for N-fixation of a range of New Zealand white clover cultivars was 1.2% per year. They concluded that the increase in N-fixation was possibly the result of indirect selection for forage yield traits. In the current study, ^15^N analysis was not performed on the *Rhizobium* treatments since the only source of N, other than an initial trace amount, was obtained from the atmosphere through the legume-TA1 *Rhizobium* symbiosis. It maybe that selection for yield traits in the field could lead to indirect selection for increased N-fixation, but this is difficult to assess, especially if the selection is undertaken on soils high in mineral N. This is supported by the current study where, as described previously, N-supplementation may confound analysis of symbiotic traits.

The predicted genetic gains per cycle for the symbiotic traits at 5% selection pressure were similar to values for white clover agronomic traits such as stolon number (~11%) and stolon branching (15–17%) reported in a recent field trial study using deterministic modelling based on 200 half-sib families (Ehoche et al., 2022). Predicted genetic gain for DM yield in that field trial, however, was lower (8.75%) and likely reflects the phenotypic complexities and G × E interactions of multi-site, multi-year field trial data. It may also be due to the population being derived from elite material previously selected for DM yield and persistence across a range of environments (Ehoche et al., 2022), whereas stolon parameters were not target traits themselves. The half-sib families used in the current symbiotic trial were a subset of the 200 assessed by Ehoche et al. (2022). The high predicted genetic gains for symbiotic traits compared with the lower gains for field-based DM may reflect the influence of controlled environment rooms. These rooms likely enabled more accurate estimation of the symbiotic trait variance components. However, the symbiotic traits had similar predicted genetic gains as the field stolon traits which may indicate that neither symbiotic nor stolon traits have been selection targets in this elite material. This suggests unexplored opportunities for symbiotic trait improvement.

Interestingly, there was some overlap between the top performing 10% (n = 20) families for field trial multi-site DM yield across years identified by Ehoche et al. (2022) and the top-ranked Shoot DM families in the current study. Two of the top performing field trial half-sib families (164, 187) comprised a third of the top-ranked 5% of families for the symbiotic traits Shoot DM and SH index. Similarly for Shoot SP, the top 10% (n = 12) of families had three (76, 164, 187) in common with top-ranked field trial families. Notably, half-sib family 187 was a strong performer in both the field trial and the symbiotic assessment across a range of traits. This suggests this family has genetic potential for enhanced DM performance across a range of environments. While it is difficult to make comparisons between multi-year multi-site field trial performance and seedling performance in symbiosis with *Rhizobium*, the commonality of some top ranked lines at higher selection pressures indicates performance at the seedling stage under symbiosis may give some insight into long-term field performance.

The Shoot DM variation among the 120 half-sib families in this study highlighted that many families, while derived from broadly-adapted elite material, did not form effective symbioses with *Rhizobium* strain TA1, a white clover commercial inoculant. Combined with the predicted genetic gains based on among-family selection, these insights indicate potential to breed for enhanced performance under symbiosis and tailor clover genetics to major commercial *Rhizobium* strains for enhanced seedling growth and establishment. This can be improved further, as a key feature of the half-sib family structure is only ¼ of the total additive variation is evaluated (Falconer and Mackay, 1996), hence among-family phenotypic selection strategies can only utilise this portion of the additive variation (Casler and Brummer, 2008). Additional increases in genetic gain for a trait can be made by accessing the ¾ additive variation residing within the half-sib families. Methodologies, such as genomic selection, which has been applied in forages (Faville et al., 2018, 2020, 2021; Ehoche et al., 2021), address this issue. The population in the current study could, therefore, provide a platform for testing genomic selection for improving genetic gain of symbiotic traits.

### Comparison of selection approaches

Single and multi-trait among-family selection methods were compared focusing on above ground yield traits Shoot DM and SP to identify half-sib families for crossing. While breeding candidates for single traits were determined by ranking half-sib families using BLUP values, this study also examined two multi-trait selection procedures: pattern analysis and the Smith-Hazel (SH) index. Although based on different methodologies, there was a high degree of commonality in half-sib families selected using the multi-trait procedures, but detailed comparison of the two methods is difficult. This is because pattern analysis is a diagnostic tool based on a graphical representation of association among all the traits assessed, hence family selection is influenced by the breeder’s interpretation of the biplot. The SH index, by contrast, is a predictive assessment tool based on estimates of additive and genetic correlations for the specific traits of interest (Smith, 1936; Hazel, 1943), and allows breeders to rank all families for multi-trait performance using a numerical index. This was, therefore, our preferred method for developing a plant ideotype with both high Shoot DM and Shoot SP by identifying half-sib families with the highest estimated genetic worth for both traits combined.

When comparing the SH selection with the single-trait selections for Shoot DM and Shoot SP, there was commonality of half-sib families. This was not unexpected given the moderate genetic and phenotypic correlations between the Shoot DM and SP. Of the top-ranked six families selected for each trait or the SH index at the highest selection pressure, five were common to Shoot DM and the SH index selections, and only two common to Shoot SP and SH index selections. This suggests that at higher selection pressures, Shoot DM is a greater contributor to the SH index than Shoot SP, and likely reflects phenotypic and additive genetic variances. The SH index has been used successfully for breeding crop species such as barley (*Hordeum vulgare*) (Eshghi et al., 2011), wheat (Gebre-Mariam and Larter, 2006) and maize (*Zea mays*) (Bänziger and Lafitte, 1997) and the biofuel crop switchgrass (*Panicum virgatum*) (Jahufer and Casler, 2015). There are, however, few examples of the SH index being applied in forage breeding, although it has been used with quality and biomass traits to identify perennial ryegrass populations with good genetic potential for cultivar development (Humphreys, 1995).

The likely success of a multi-trait selection, such as the SH index, for Shoot DM and Shoot SP is also supported by the 11–13% correlated response to selection for these two traits. Even so, while selecting for Shoot DM will also increase Shoot SP, not all the highest Shoot DM plants will have a high SP. Implementation of a SH index will assist in facilitating combined improvement of these two traits. Correlated responses to selection have been investigated for a range of white clover seed, stolon and yield traits (Rowe and Brink, 1993; Annicchiarico and Piano, 1997; Collins et al., 1997). There is, however, limited information available regarding correlated response to selection for symbiotic traits in white clover. One study noted that selection for increased nodule mass resulted in a correlated response of increased nodule number per plant (Jones and Burrows, 1968).

There are advantages and disadvantages for single or multi-trait selection schemes. Single-trait selection can achieve higher rates of genetic gain per cycle per trait compared to a multi-trait approach. The results of this study demonstrate application of single and multi-trait selection indices, such as the SH index, for facilitating the ranking of half-sib families in future symbiotic work. The half-sib families selected across these indices could provide the basis of proof-of-concept crosses for among-family breeding, that may also be enhanced by genomic selection, to improve white clover-*Rhizobium* symbiotic agronomic traits.

### Conclusion and breeding applications

To our knowledge, this is the first study to detail a quantitative genetic analysis of white clover-*Rhizobium* symbiosis in a half-sib family population. The significant family additive genetic variation ($\sigma_{A}^{2}$) for symbiotic traits, moderate narrow-sense heritabilities ($h_{n}^{2}$) and predicted genetic gains at different selection pressures indicate potential for breeding white clover seedlings with enhanced interaction with TA1, a commercial *Rhizobium* inoculum strain. The half-sib family population used in the trial was derived from broadly-adapted, top performing breeding lines, yet the variation in Shoot DM and SP under symbiosis demonstrated many of the families were poorly compatible with the TA1 strain. Such variation indicates that further consideration of symbiotic competency may be needed during the development of this material, highlighting a breeding opportunity. This was supported by the high levels of predicted genetic gain per cycle for symbiotic traits. Furthermore, the moderate positive phenotypic correlation (*r* = 0.47) between Shoot DMs with either supplemental mineral N or under symbiosis indicated N-supplementation was not an accurate predictor of symbiotic performance and may confound analysis of symbiosis-based traits. This suggests that symbiotic traits themselves should be the focus for improving interactions with *Rhizobium*.

A breeding target based on an ideotype aligning high biomass with efficient use of symbiotically-derived N requires integration of high Shoot DM and high Shoot SP. The SH index would be an efficient method to facilitate this process, particularly given the moderate genetic correlation between the traits and the commonality of families in the top-ranked selections of the two individual traits and the SH index. Genetic gain will likely be enhanced through application of genomic selection in this half-sib family population. Additionally, the strong genetic correlations between shoot and root symbiotic traits indicate strong pleiotropy or linkage among genetic mechanisms for these features. This suggests that if above ground traits are the target, then there is reduced value in measuring root traits, freeing resources for use elsewhere.

In this study, a single *Rhizobium* strain was assessed in a controlled environment to reduce the G_Plant_ × G*_Rhizobium_* × E interactions, thus increasing ability to estimate variance components. Implementation of these insights for enhanced symbiosis in the field could be through aligning clover genetics to a commercial *Rhizobium* strain applied as an inoculum coating prior to sowing. Using this approach as a vehicle for improved white clover establishment without requirement of N-fertiliser application will reduce farm-system inputs and has implications for other forage and annual grain legume crops. Moving beyond seedling establishment to investigating symbiotic potential of perennial mature plants in the field will require further investigation. An avenue to explore includes developing methods to provide the commercial *Rhizobium* strain as a regular pasture application throughout the year to the aligned white clover genetics as the plants mature and transition to the perennial creeping stoloniferous form. The current study can also be extended to assess clover germplasm with soil-derived *Rhizobium* populations as a basis for identifying clover germplasm with a wider effective symbiotic compatibility. In the near future, the half-sib population used in this study and the data generated provide a platform to breed white clover with improved symbiotic traits, and may be regarded as a training population to explore genomic selection for improvement of these complex plant-microbial interactions.

## Data availability statement

The original contributions presented in the study are included in the article/Supplementary material, further inquiries can be directed to the corresponding authors.

## Author contributions

SW: data curation, formal analysis, investigation, methodology, visualisation, writing – original draft, and writing – review and editing. MJ: formal analysis, visualisation, writing – original draft, and writing – review and editing. RH: supervision and writing – review and editing. CA: data curation, investigation, and methodology. DL: formal analysis. OE: investigation and resources. GC: resources. EJ: supervision and writing – review and editing. RB: supervision, writing – original draft, and writing – review and editing. AG: conceptualization, project administration, investigation, supervision, visualisation, writing – original draft, and writing – review and editing. All authors contributed to the article and approved the submitted version.

## Funding

This work was conducted within the New Zealand Ministry of Business, Innovation and Employment (MBIE) grant C10X1308 ‘Improving Legume-Rhizobia Performance’. Additional funding was provided by the Lincoln University, Agriculture and Life-Science Faculty post-graduate fund.

## Conflict of interest

OE and GC were employed by PGG Wrightson Seeds Ltd.

The remaining authors declare that the research was conducted in the absence of any commercial or financial relationships that could be construed as a potential conflict of interest.

## Publisher’s note

All claims expressed in this article are solely those of the authors and do not necessarily represent those of their affiliated organizations, or those of the publisher, the editors and the reviewers. Any product that may be evaluated in this article, or claim that may be made by its manufacturer, is not guaranteed or endorsed by the publisher.

## Footnotes

^1^ http://agrubuntu.cloudapp.net/PlantBreedingTool/
