# Supplementary material for: Quantitative genetic analysis reveals potential to breed for improved white clover growth in symbiosis with nitrogen-fixing Rhizobium bacteria
Source: Front Plant Sci. 2022 Sep 20;13:953400. doi: 10.3389/fpls.2022.953400 (PMC9534031; doi:10.3389/fpls.2022.953400)
Supplement: Supplementary file 1 [file Data_Sheet_1.docx]

Supplementary Material

# Supplementary Figures and Tables

## Supplementary Figures


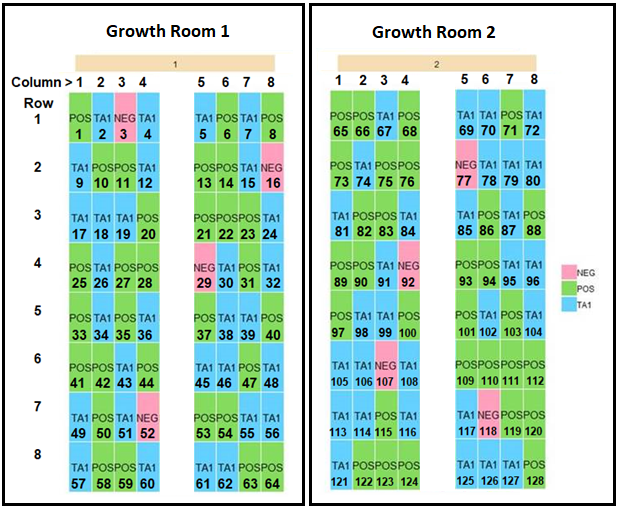


Supplementary Figure S1: Randomised split plot experimental design used for screening 120 half-sib families. Treatment containers were numbered from 1 to 128 consisted of half-sib families inoculated with the *Rhizobium* *leguminosarum* bv. *trifolii* strain TA1 (blue) or grown under mineral nitrogen (N) (positive control) (green). Each half-sib family was represented by 16 individuals per container per growth room resulting in 32 biological replicates per family across the two Growth Rooms. Containers labelled in red were negative controls sown with a bulked mixed sample of all half-sibling families that were not inoculated with *Rhizobia* nor provided with mineral N. Each container was considered a plot and partitioned into four quadrants (sub-plots) with one randomly assigned half-sibling family per quadrant. Containers were randomised across two separate growth rooms (environmental replicates) using a row and column experimental design.


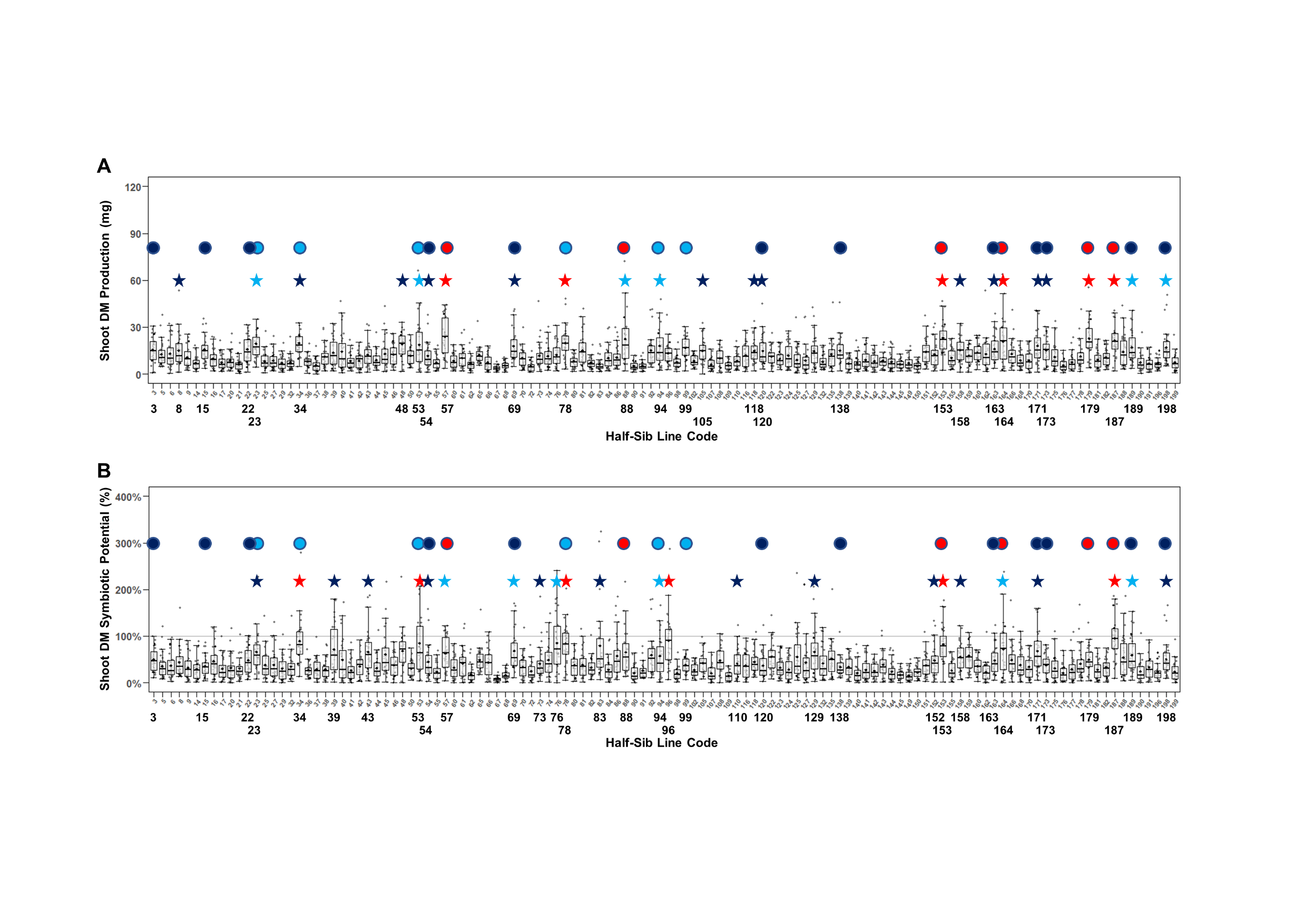
**Supplementary Figure S2:** Selected half-sib family lines and their trait variation in **(A)** Shoot dry matter production and **(B)** Shoot DM symbiotic potential of 120 white clover half-sib lines 35 days after inoculation with *Rhizobium leguminosarum* bv. *trifolii* strain TA1. Each half-sib family was represented by 32 biological replicates with 16 in each growth room. Circles and stars represent top ranked half-sib families selected using either the multi-trait Smith-Hazel index or the individual trait BLUPs, respectively. Red indicates selected half-sib families at 5% selection pressure (n = 6); combining Red + Light Blue indicates selected half-sib families at 10% selection pressure (n = 12); and Red + Light Blue + Dark Blue indicates selected half-sib families at 20% selection pressure (n = 24).

## Supplementary Tables

Supplementary Table S1: Pearson’s pairwise phenotypic correlation plot of five traits of 120 white clover half-sibling families inoculated with a pure culture of the *Rhizobium leguminosarum* bv. *trifolii* strain TA1 or grown with no rhizobia and mineral nitrogen (POS). Data representing traits are presented as best linear unbiased predictor (BLUP) adjusted means. Plant traits are: Shoot DM (dry matter plant^‑1^); Root DM; RSR (root to shoot ratio plant^‑1^); Shoot SP (symbiotic potential plant^‑1^); and Root SP. The bold text, size and colour intensity of the circle are proportional to the correlation coefficient (*r*). Positive correlations are displayed in white-red and negative correlations in yellow-green colour. Numbers in the lower triangle with white background are *P* values which indicate significant association at *P*≤0.05.


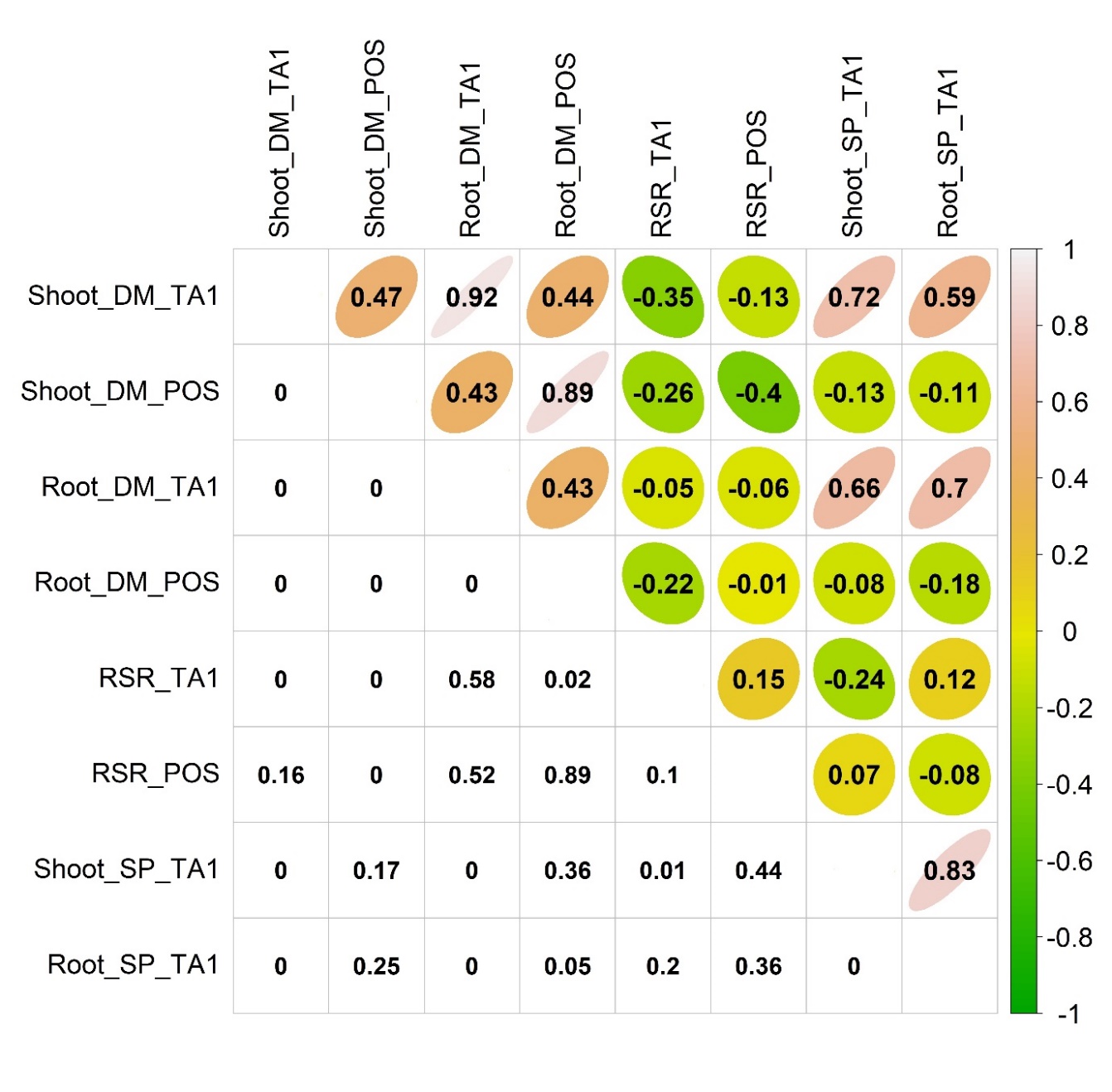


Supplementary Table S2: White clover half-sib families selected at selection pressures of 20%, 10% and 5% per selection cycle from 120 half-sib families inoculated with the *Rhizobium leguminosarum* bv. *trifolii* strain TA1 based on their breeding values (BLUPs) for the symbiotic traits Shoot dry matter (DM) and Shoot symbiotic potential (SP) or Shoot DM and Shoot SP BLUPs adjusted for the Smith-Hazel index (SH index).

|  | | |  | Single-trait selection | | | | Multi-trait selection | |
| --- | --- | --- | --- | --- | --- | --- | --- | --- | --- |
| Selection pressure | | | **Rank** | ***Half-sib Family no.*** | **Shoot DM BLUP value (mg)** | ***Half-sib Family no.*** | **Shoot SP BLUP value (%)** | ***Half-sib Family no.*** | **SH index value** |
| 20% | **10%** | **5%** | **1** | ***57*** | 23.3 | ***187**** | 85 | **57†** | 13.1 |
|  |  |  | **2** | ***187**** | 20.5 | ***78*** | 82 | 179^1^**†** | 12.7 |
|  |  |  | **3** | ***153*** | 20.3 | ***153*** | 72 | 88^1^ | 12.2 |
|  |  |  | **4** | ***78*** | 20.1 | *96* | 71 | **153†** | 11.9 |
|  |  |  | **5** | *179^1^* | 19.7 | ***34*** | 70 | **164†*** | 11.3 |
|  |  |  | **6** | ***164**** | 19.6 | ***53*** | 70 | **187†*** | 10.6 |
|  |  |  | **7** | ***94*** | 18.9 | *76****** | 69 | **23†** | 10.6 |
|  |  |  | **8** | ***53*** | 17.6 | ***164**** | 65 | 48^1^ | 10.4 |
|  |  |  | **9** | *88^1^* | 17.4 | ***57*** | 64 | **94†** | 10.2 |
|  |  |  | **10** | ***198*** | 17.3 | ***94*** | 62 | **78** | 10.1 |
|  |  |  | **11** | ***23*** | 16.9 | ***69*** | 60 | 99 | 10.0 |
|  |  |  | **12** | ***189*** | 16.9 | ***189*** | 60 | **34†** | 9.97 |
|  |  |  | **13** | *105* | 16.9 | *83* | 59 | **171** | 9.79 |
|  |  |  | **14** | ***69*** | 16.3 | *39* | 58 | **198†** | 9.72 |
|  |  |  | **15** | ***171*** | 16.3 | *129* | 58 | **69†** | 9.72 |
|  |  |  | **16** | *8* | 16.0 | *54* | 58 | **53†** | 9.69 |
|  |  |  | **17** | ***34*** | 15.9 | ***171*** | 57 | 15***** | 9.60 |
|  |  |  | **18** | *173^1^* | 15.8 | ***23*** | 55 | **189†** | 9.46 |
|  |  |  | **19** | *48^1^* | 15.6 | *43* | 54 | 163^1^ | 9.42 |
|  |  |  | **20** | *120^1^* | 15.6 | *110* | 54 | 22***** | 9.26 |
|  |  |  | **21** | *54* | 15.5 | *73* | 52 | 173^1^ | 9.22 |
|  |  |  | **22** | *118* | 15.5 | *158* | 52 | 138 | 9.21 |
|  |  |  | **23** | *158* | 15.4 | ***198*** | 52 | 3 | 9.15 |
|  |  |  | **24** | *163^1^* | 15.2 | *152* | 52 | 120^1^ | 9.07 |

Families in **bold** are common to all three selections; underlined are common to both single trait selections; superscript^1^ common to Shoot DM and Multi-trait selections; †common to SH index and Pattern analysis; and the remainder are in a single selection. *common to top ranked 20% field trial half-sib families (Ehoche et al., 2022 (*in press*) doi: 10.1002/csc2.207983)
